# Recurrence of Varicose Vein after Endovenous Laser Therapy in a Tertiary Care Center: A Descriptive Cross-sectional Study

**DOI:** 10.31729/jnma.6163

**Published:** 2021-03-31

**Authors:** Dinesh Chapagain, Kiran Prasad Shrestha, Deepak Thapa Magar, Kumar Bahadur Shrestha, Pramod Kumar Yadav

**Affiliations:** 1 Department of Cardiothoracic and Vascular Surgery (CTVS), National Academy of Medical Sciences, Bir Hospital, Mahabauddha, Kathmandu, Nepal

**Keywords:** *laser ablation*, *recanalization*, *varicosity*

## Abstract

**Introduction:**

Varicosity is the common problem of various etiology having simple limb aching to worst complications like oedema, ulcer, and skin changes. Minimal invasive endovenous laser therapy is a noble procedure. The aim of the study is to find out the recurrence of the varicose vein after laser therapy in a tertiary care center.

**Methods:**

This descriptive cross-sectional study was done in 38 patients with varicosity of the lower limb in a tertiary care hospital, from January 2019 to June 2019 after taking ethical clearance from Institutional Review Committee. Convenience sampling was done. Data was collected and entry was done in Statistical Package for the Social Science software version 22, point estimate at 90% Confidence Interval was calculated along with frequency and proportion for binary data.

**Results:**

We recorded 38 patients with ablated limb out of which none of the ablated veins showed recanalization in six months follow up. Twenty two (58%) patients were male and 16 (42%) patients were female with a mean age of 40.26 years. Major bulk, 23 (60.5%) resumed activity in second postoperative day and only 1 (2.6%) patient waited for 5 days for normal activity with mean of 2.58 days postoperatively. Sixteen (42.1%) patients developed erythema or ecchymosis, 12 (31.6%) patients had induration along the long saphenous vein course, 7 (18.4%) patients had paresthesia, 2 (5.3%) patients had limb swelling and 1 (2.6%) patient had skin burn.

**Conclusions:**

Endovenous laser ablation has very low rate of recurrence of varicosity and has minor complications.

## INTRODUCTION

Varicosity is defined as abnormal dilatation and tortuosity of the lower limb superficial veins, presented as aching, heaviness, edema, skin pigmentation, bleeding, chronic ulcer and mostly asymptomatic. Open surgical method is standard procedure of choice.

The main rationale of the study of the endovenous procedure is minimal invasive and lower complication rate. Ultrasound-guided endovenous laser ablation, foam sclerotherapy and radiofrequency ablation are all consistently proving to be at least as beneficial as surgery, without the same complications and with less post-procedure morbidity and more rapid recovery.^1^

Endovenous laser ablation eliminates reflux with less morbidity, faster recovery, and improved cosmetic results with high patient satisfaction. As such, it has become the preferred treatment method for varicose veins since it was first introduced a decade ago.^2^

The aim of the study is to see the recurrence of varicose vein after endovenous laser therapy in a tertiary care center.

## METHODS

This descriptive cross-sectional study conducted from January 2019 to June 2019 for 6 months at National Academy of Medical Sciences, Bir hospital in the department of cardiothoracic and vascular surgery. Convenience sampling method was used. The cases of deep vein thrombosis and pregnancy were excluded from the study.

Sample size was calculated using the formula,

$$\begin{array}{ll} \text{n} & ={\text{Z}}^{\text{2}}\times \text{p}\times \text{q}/{\text{e}}^{\text{2}}\\ & ={\left(1.64\right)}^{2}\times (0.13)\times (1-0.13)/{\left(0.1\right)}^{\text{2}}\\ & =30.41 \end{array}$$

Where,

n = required sample size,
Z = 1.64 at 90% Confidence Interval
p = prevalence of recurrence, 13%^3^
q = 1-p
e = margin of error, 10%

The calculated minimum sample size was 31 and 38 patients were taken in our study. Every patient included in this study were examined by concerned surgeon and anesthetics for fitness for spinal anesthesia after taking consent for endovenous laser therapy. All the patients were taken to operation theatre and spinal anesthesia were given and great saphenous vein were ablated with 400nm fiber using diode laser with 10-watt power with 80 joule energy in each pulse for each cm segment of vein by adjustment of tip of the fiber just 1 cm from the saphenofemoral junction. While applying laser patient was kept in trendelenberg position for the aim of emptying the vein and perivenous region were cooled by normal saline till just below knee level which is the distal end point of ablation. During ablation extra cooling done by using ice packed wrapped in tetra and applied over the ablation site to prevent superficial burn. Varicosity below the knee were obliterated by 2% sodium tetradecayl sulphate (STD) which were made as foam by mixing with 4 ml of air and 1ml of STD and injected into the varicose slowly after evacuating the vein. Maximum 4ml of foam agent used in single patient to prevent from embolization. Then crepe bandage applied over the limb. This procedure was done under the guidance of USG color Doppler. Patients were shifted to post up where single dose of 40mg cutena subcutaneously (low molecular weight heparin) were given in the evening and next dose to be given next morning during discharge. USG Color Doppler were regularly used at the end of ablation to record reflux from deep vein especially from saphenofemoral junction. Then the patients were discharged with simple analgesics and aspirin 75mg for 15 days to prevent thrombosis. Patients were adviced to visit to Out Patient Department (OPD) after 1 week, at 1 month and 6 months. At each OPD follow up, venous Doppler were done in CTVS department for recurrence of saphenofemoral junction reflux. Other complications and resume of normal activity on the basis of questionnaire on what date the patients can resume outdoor activities as before were also recorded. Data was collected and entry was done in Statistical Package for the Social Science software version 22, point estimate at 90% Confidence Interval was calculated along with frequency and proportion for binary data.

## RESULTS

**Figure 1.**
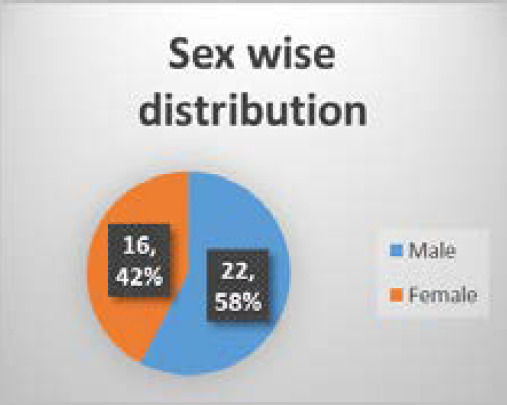
Sex wise distribution.

**Figure 2.**
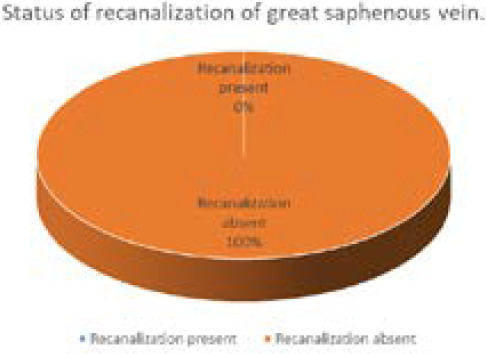
Recanalization of the ablated vein

**Figure 3.**
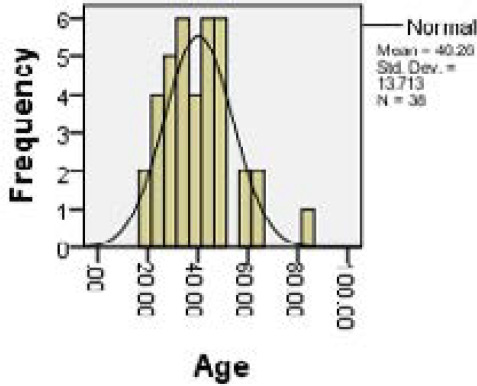
Age distribution.

We recorded 38 patients of ablated limb in which none of the ablated veins showed recanalization in six months follow up. Twenty two (58%) patients were male and 16 (42%) patients were female with mean age of 40.26 years and standard deviation 13.71 (Figure 1, 2 and 3). All the patients had undergone endovenous laser ablation surgery starting from just 1 cm below saphenofemoral junction to just below the knee. There were not any recanalization in six month of follow up (Figure 2). During the postoperative days 16 (42.1%) patients developed erythema or ecchymosis, 12 (31.6%) patients had induration along the long saphenous vein course, 7 (18.4%) patients complained of paresthesia mostly around knee area, 2 (5.3%) patients had limb swelling and 1 (2.6%) patient had skin burn around knee region (Table 1).

**Table 1:** Complication after EVLT.

Complication	n (%)
Induration	12 (31.6)
Erythema/Ecchymosis	16 (42.1)
Skin burn	1 (2.6)
Swelling	2 (5.3)
Paresthesia	7 (18.4)

Twenty three (60.5%) resumed normal activity in second postoperative day, 9 (23.7%) patients start normal activity in 3rd postoperative days, 5 (13.2%) patients resumed normal activity on 4th postoperative day and only 1 (2.6%) patients waited for 5 days for normal activity with mean of 2.58 days postoperatively (Table 2).

**Table 2:** Physical Activity after EVLT.

Post operative days	n (%)
2	23 (60.5)
3	9 (23.7)
4	5 (13.2)
5	1 (2.6)

## DISCUSSION

In demographic profile 42.1% were female and 57.9% male with age ranges from 19 years to 82 years which has similarity with the male predominance shown from university of edinburg of cross-sectional survey but in another study in new guinea showed the very low prevalence in female.^4,5^ Chaar CI also showed very low 1.6% recanalization rate of great saphenous vein out of 885 limbs.^6^ Kenneth myers et al. showed 80% primary success rate in 404 veins in 3 years follow up.^7^ LS Alder et al. had 99% success rate in two months follow up time which has similar features with our study.^8^ Complete success rate in our study may be because of small number of patients or may be because of very early period after surgery. This minimal invasive surgery mainly done under local anesthesia or spinal anesthesia, ambulate at the same day of surgery.^9,10^ We also performed endovenous laser surgery under spinal anesthesia and mostly mobilized by the evening when it was performed early in the morning and discharged on next day early morning when performed in the afternoon. As this is the minimal invasive procedure where wound is very less likely. Patients resume normal daily activity in a short period of time after surgery. One of the studies by Geneva university showed the mean of 6.9 days for the return to normal activity.^11^ In our study the mean of 2.56 days to return to normal activity which is in contrast to some other study above. Another study by Osman abu-elcibba had shown average 4.8 days to return to normal activity.^12^ Which is roaming around home with comfort and doing house hold activity. Some of the minor complications are expected. Most of the patients 42.1% had erythema or ecchymosis along the course of long saphenous vein with 31.6% complained of induration. Other few patients complained of paresthesia, swelling of limb and superficial burn. In similar study by Desmyttere J et al. had also majority of ecchymosis with paresthesia of 7% without any major complication as DVT and PE as in our study.^13^ One of the review article described complication in various ranges like ecchymosis 2.75% - 100%, paresthesia 3% - 41%, burns 0-1.3%, with DVT and PE less than 0.5% having similarity to our study.^14^ The major limitations of this study are small sample size, short term, single institutional study and convenient sampling method. Because of these limitations, though endovenous therapy can be the treatment of choice in respect to recurrence and better quality of life, may need more extensive study to generalize the finding.

## CONCLUSIONS

Endovenous laser ablation has very low rate of recurrence of varicosity and has minor complications.
